# Subdural and epidural hematoma following lumbar puncture in patients on anticoagulants or antiplatelets: A retrospective propensity score-matched registry study

**DOI:** 10.1007/s13760-026-03019-7

**Published:** 2026-03-23

**Authors:** Jinpyo Hong, David R. Hallan, Mason T. Stoltzfus, Manuel Bita, Kunal Koka, Elias Rizk

**Affiliations:** 1 Department of Neurosurgery, Penn State Health Milton S. Hershey Medical Center, Hershey, PA 17033 USA; 2 Georgia State University, Atlanta, GA 30302 USA; 3 Department of Neurosurgery, Dell Children’s Hospital, The University of Texas at Austin, Austin, TX 78723 USA

**Keywords:** Subdural, Epidural, Hematoma, Lumbar puncture, Anticoagulant, Antiplatelet

## Abstract

**Background:**

Lumbar punctures are one of the most used diagnostic and therapeutic procedures for a range of CNS pathologies, including infection and increased intracranial pressure due to many etiologies. Lumbar punctures violate the dura of the thecal sac, posing some risk for the formation of epidural or subdural hematomas. This risk may be increased in patients on anticoagulant or antiplatelet therapy. This study compares the odds of developing epidural or subdural hematomas, paralysis, and the requirement of epidural blood for hemostasis within 30 days of lumbar puncture in patients with and without anticoagulant or antiplatelet use in hopes of establishing evidence-based guidelines for clinical decision-making for lumbar punctures in patients using these medications.

**Methods:**

The TriNetX multi-institutional electronic health record database was used to perform a retrospective, propensity score-matched analysis of outcomes of cohorts of patients who underwent lumbar punctures on anticoagulants, antiplatelets, and no anticoagulants or antiplatelets within one month of the procedure. The outcomes of interest were examined within 30 days of lumbar puncture and included the occurrence of epidural or subdural hematoma, paralysis, requirement of an epidural blood patch, and requirement of spinal decompression.

**Results:**

A total of 25,788 patients were identified for both lumbar puncture patients with anticoagulants and without blood thinners after propensity score matching. For this study, epidural hematoma (OR [95%CI], 1.000 [0.561–1.784]) showed no difference in odds ratio between the two cohorts, while subdural hematoma (OR [95%CI], 0.780 [0.640–0.950]) showed a statistically significantly lower odds ratio for the anticoagulant use group. We identified 13,310 patients for both lumbar puncture patients with antiplatelets and lumbar puncture patients without antiplatelet, anticoagulant, heparin, and LMWH (“blood thinners”) after propensity score matching. For this analysis, epidural hematoma (OR [95%CI], 1.904 [0.885–4.095]) showed a higher odds ratio compared to subdural hematoma (OR [95%CI], 1.008 [0.784–1.296]), both of which were not statistically significant.

**Conclusion:**

The data analyses suggest that there are no increased risks of epidural and subdural hematoma in patients with a history of use of antiplatelet or anticoagulant compared to those who do not. These results could be further validated by a single or multi-institutional retrospective cohort study with additional parameters that characterize the patient population.

## Introduction

Lumbar puncture is a commonly used invasive test used to diagnose and treat headaches, inflammatory diseases, and infectious diseases of the nervous system [1]. Diagnostics using lumbar punctures involve collecting cerebrospinal fluid samples for lab testing [2]. An example of an infectious disease of the nervous system whereby lumbar puncture diagnostics are essential is meningitis [3]. Lumbar puncture procedures are relatively safe as complications rarely arise [4]. Although relatively rare, lumbar puncture procedures are associated with complications such as spinal hematomas, pain at the puncture site, dizziness, and post-lumbar headache (PLPH). The most common complication observed is PLPH, with an incidence of 32% [1]. Spinal hematoma, which is an accumulation of blood in the spinal canal, is among the most critical complications associated with lumbar puncture procedures [3, 5]. Spinal hematomas typically lead to a compression of the spinal cord, leading to various neurological deficits [6]. A widely used remedy for that is spinal decompression, which is a conservative treatment method for intervertebral disc herniation. Spinal decompression works by reducing pressure on the spinal cord by supplying nutrients and oxygen to the disc [7]. There exist three main types of spinal hematomas: subdural, epidural, and subarachnoid hematomas [6]. A spinal subdural hematoma, also called spinal subdural hemorrhage, is an accumulation of blood in subdural space, which is the space between the spinal arachnoid and the dura membrane [8]. A spinal epidural hematoma is a collection of blood between the spinal dura mater and the surface of the vertebrae. Spinal hematomas are relatively rare neurosurgical emergencies, and when they occur, they are often fatal, with a death rate of about 50% [9]. 

Anticoagulants are widely used drugs that prevent blood coagulation or clotting. They are mostly used in venous thromboembolism (VTE) treatment [10]. Antiplatelets, on the other hand, help prevent the accumulation of platelets, which is an important pathophysiological aspect of vascular accidents such as hematomas [11]. Understanding the dearth of literature in the realm of understanding how the history of use of anticoagulants or antiplatelets affects the occurrence of subdural and epidural hematomas, this study investigates the risk of developing subdural and epidural hematoma following lumbar puncture for patients with a history of antiplatelet and anticoagulant use. We hypothesize that a history of use of both anticoagulants and antiplatelets would increase the risks of developing subdural and epidural hematomas [12]. 

## Methods

### Study design

This retrospective cohort study used the TriNetX Research Network database, which includes data from over 106 million patients from 68 healthcare networks across the world, to query for patients who underwent lumbar punctures with and without anticoagulant and antiplatelet use using the International Classification of Diseases, 10th Revision (ICD-10), current procedural terminology (CPT) codes, RxNorm, and VA formulary medication systems. TriNetX is a de-identified patient database that uses electronic medical records from multiple institutions worldwide. No Institutional Review Board (IRB) approval or patient consent was required due to the de-identified nature of the database. Previous literature confirmed the utility and validity of this database, and its details are well-described [13–15]. 

### Patient selection

The TriNetX database we queried on September 25, 2022, for patients of any age and included outcomes from LPs with and without anticoagulant or antiplatelet use that occurred within the past 20 years. Patients that underwent lumbar punctures were selected using CPT codes 00635, 62,270, 1,035,653, and 62,328 and ICD-10 codes 009U3ZX, 009U3ZZ, 009U30Z, 009U4ZX, 009U40Z, and 009U4ZZ. A control cohort included any patients who underwent any of the aforementioned LP procedures and were not exposed to anticoagulants or antiplatelets. Patients in the anticoagulant-receiving cohort were further classified as having been exposed to enoxaparin, heparin, and other anticoagulants using RxNorm codes 67,108 and 5224, along with VA formulary code BL110, respectively. Patients in the antiplatelet cohort were selected using RxNorm codes 32,968, 10,594, 1191, 1,116,632, 3521, and 613,391 for exposure to clopidogrel, aspirin, prasugrel, and more. Patients who received protamine sulfate, idarucizumab, platelet transfusions, prothrombin, blood, or blood components were excluded due to the potential reversal of anticoagulation.

Using CPT codes, the resulting patient cohorts were examined for covariates that may confound results, including thrombocytopenia, Von Willebrand’s disease, platelet defects, age, sex, demographics, and more. The cohorts were then propensity score-matched in an attempt to mimic true randomization.

### Statistical analysis

Various statistical tools, including risk difference (formerly referred to as attributable risk), odds ratio (OR), risk ratio (RR, also known as relative risk), and standard mean difference (SMD), were used to analyze data. Risk difference is helpful for quantifying the difference in risk of experiencing an outcome between exposed and unexposed cohorts. The risk ratio quantifies the ratio of the risk of experiencing an outcome between exposed and unexposed cohorts. The odds ratio quantifies the odds of experiencing an outcome between exposed and unexposed cohorts [16]. SMD quantifies the difference between cohorts, where a value of zero indicates no difference between cohorts [17]. An alpha level of 0.05 was used to determine statistical significance. Data was analyzed using TriNetX software with JAVA, R, and Python programming languages.

Outcomes were examined within 30 days of LP and included the occurrence of epidural or subdural hematoma (EDH and SDH, respectively), paralysis, requirement of an epidural blood patch (EBP), and requirement of spinal decompression. The 30-day time-limit excludes complications unrelated to LP.

## Results

Before propensity score-matching, we identified 26,704 lumbar puncture patients with anticoagulant use history and 165,605 lumbar puncture patients without antiplatelet, anticoagulant, heparin, and LMWH (“blood thinners”) use. Age at index were 48.8 ± 22.2 years and 28.5 ± 23.2 years for anticoagulant-exposed and unexposed cohorts, respectively. 47.8% of the anticoagulant-exposed cohort and 44.5% of non-anticoagulant-exposed cohort were males.

Pre-propensity score-matching baseline characteristics and demographics are shown in Table 1 for the anticoagulant versus non-anticoagulant comparative study.

**Table 1:** Pre-propensity score-matching baseline characteristics and demographics of lumbar puncture patients with and without anticoagulant use history

Baseline characteristic	Anticoagulant-exposed	% of cohort	Non-anticoagulant-exposed	% of Cohort	*p*-value	Standard mean difference
Patients	26,704	100%	165,605	100%		
Age at Index	48.8 (+/- 22.2 SD)	100%	28.5 (+/- 23.2 SD)	100%	< 0.001	0.894
Male	12,756	47.8%	66,381	44.5%	< 0.001	0.066
Female	13,905	52.2%	82,666	55.5%	< 0.001	0.066
White	16,756	62.8%	92,751	62.2%	0.053	0.013
Black or African American	5,356	20.1%	25,216	16.9%	< 0.001	0.082
Asian	567	2.1%	2,798	1.9%	0.006	0.018
Unknown Race	3,771	14.1%	27,230	18.3%	< 0.001	0.112
Fibrosis and Cirrhosis of Liver	533	2.0%	945	0.6%	< 0.001	0.120
Hypertensive Diseases	10,741	40.3%	20,179	13.5%	< 0.001	0.633
Diabetes Mellitus	4,958	18.6%	8,065	5.4%	< 0.001	0.414
Overweight, Obesity, Other Hyperalimentation	3,936	14.8%	10,279	6.9%	< 0.001	0.255
Nicotine Dependence	4,322	16.2%	11,080	7.4%	< 0.001	0.274
Alcohol Abuse	1,229	4.6%	2,131	1.4%	< 0.001	0.187
Alcohol Dependence	1,184	4.4%	1,745	1.2%	< 0.001	0.199
Chronic Lower Respiratory Diseases	4,145	15.5%	12,992	8.7%	< 0.001	0.210
Atrial Fibrillation and Atrial Flutter	1,810	6.8%	1,497	1.0%	< 0.001	0.302
Heart Failure	2,011	7.5%	2,129	1.4%	< 0.001	0.299
Ischemic Heart Diseases	3,463	13.0%	3,176	2.1%	< 0.001	0.420
Other Peripheral Vascular Diseases	906	3.4%	1,265	0.8%	< 0.001	0.178
Thrombocytopenia, Unspecified	1,906	7.1%	3,581	2.4%	< 0.001	0.224
Immune Thrombocytopenic Purpura	69	0.3%	186	0.1%	< 0.001	0.031
Qualitative Platelet Defects	50	0.2%	93	0.1%	< 0.001	0.035
Von Willebrand’s Disease	19	0.1%	97	0.1%	0.717	0.002
Hereditary Factor XI Deficiency	10	0.0%	12	0.0%	< 0.001	0.020
Disseminated Intravascular Coagulation	141	0.5%	271	0.2%	< 0.001	0.058
Hereditary Factor VIII Deficiency	10	0.0%	37	0.0%	0.243	0.007
Hereditary Factor IX Deficiency	10	0.0%	15	0.0%	0.001	0.018
Activated Protein C Resistance	107	0.4%	140	0.1%	< 0.001	0.062
Prothrombin Gene Mutation	87	0.3%	121	0.1%	< 0.001	0.054
Antiphospholipid Syndrome	107	0.4%	135	0.1%	< 0.001	0.063
Lupus Anticoagulant Syndrome	97	0.4%	131	0.1%	< 0.001	0.058

This table displays the baseline characteristics and demographics of lumbar puncture patients in the anticoagulant-exposed and unexposed cohorts before propensity score matching. An alpha level of 0.05 was selected for statistical significance

SD: standard deviation

We identified 14,395 antiplatelet-exposed lumbar puncture patients and 165,605 non-antiplatelet-exposed (including antiplatelet, anticoagulant, heparin, and LMWH (“blood thinners”)) lumbar puncture patients before propensity score-matching. Age at index were 59.2 ± 17.8 years and 28.5 ± 23.2 years for antiplatelet-exposed and unexposed cohorts, respectively. 49.8% of the antiplatelet-exposed cohort and 44.5% of the non-antiplatelet-exposed cohort were males. Pre-propensity score-matching baseline characteristics and demographics are shown in Table 2 for the antiplatelet versus non-antiplatelet comparative study.

**Table 2:** Pre-propensity score-matching baseline characteristics and demographics of lumbar puncture patients with and without antiplatelet use history

Baseline characteristic	Antiplatelet-exposed	% of cohort	Non-antiplatelet-exposed	% of cohort	*p*-value	Standard mean difference
Patients	14,395	100%	165,605	100%		
Age at Index	59.2 (+/- 17.8 SD)	100%	28.5 (+/- 23.2 SD)	100%	< 0.001	1.485
Male	7,117	49.8%	66,381	44.5%	< 0.001	0.105
Female	7,176	50.2%	82,666	55.5%	< 0.001	0.105
White	9,413	65.9%	92,751	62.2%	< 0.001	0.076
Black or African American	2,758	19.3%	25,216	16.9%	< 0.001	0.062
Asian	234	1.6%	2,798	1.9%	0.042	0.018
Unknown Race	1,774	12.4%	27,230	18.3%	< 0.001	0.163
Fibrosis and Cirrhosis of Liver	365	2.6%	945	0.6%	< 0.001	0.154
Hypertensive Diseases	8,048	56.3%	20,179	13.5%	< 0.001	1.004
Diabetes Mellitus	4,076	28.5%	8,065	5.4%	< 0.001	0.647
Overweight, Obesity, Other Hyperalimentation	2,630	18.4%	10,279	6.9%	< 0.001	0.351
Nicotine Dependence	2,323	16.3%	11,080	7.4%	< 0.001	0.276
Alcohol Abuse	680	4.8%	2,131	1.4%	< 0.001	0.193
Alcohol Dependence	624	4.4%	1,745	1.2%	< 0.001	0.196
Chronic Lower Respiratory Diseases	2,791	19.5%	12,992	8.7%	< 0.001	0.314
Atrial Fibrillation and Atrial Flutter	1,804	12.6%	1,497	1.0%	< 0.001	0.474
Heart Failure	2,075	14.5%	2,129	1.4%	< 0.001	0.498
Ischemic Heart Diseases	3,556	24.9%	3,176	2.1%	< 0.001	0.706
Other Peripheral Vascular Diseases	882	6.2%	1,265	0.8%	< 0.001	0.292
Thrombocytopenia, Unspecified	1,326	9.3%	3,581	2.4%	< 0.001	0.296
Immune Thrombocytopenic Purpura	47	0.3%	186	0.1%	< 0.001	0.043
Qualitative Platelet Defects	49	0.3%	93	0.1%	< 0.001	0.062
Von Willebrand’s Disease	10	0.1%	97	0.1%	0.827	0.002
Hereditary Factor XI Deficiency	10	0.1%	12	0.0%	< 0.001	0.031
Disseminated Intravascular Coagulation	100	0.7%	271	0.2%	< 0.001	0.078
Hereditary Factor VIII Deficiency	10	0.1%	37	0.0%	0.002	0.021
Hereditary Factor IX Deficiency	10	0.1%	15	0.0%	< 0.001	0.030
Activated Protein C Resistance	99	0.7%	140	0.1%	< 0.001	0.096
Prothrombin Gene Mutation	83	0.6%	121	0.1%	< 0.001	0.087
Antiphospholipid Syndrome	102	0.7%	135	0.1%	< 0.001	0.099
Lupus Anticoagulant Syndrome	95	0.7%	131	0.1%	< 0.001	0.094

This table displays the baseline characteristics and demographics of the patients in the antiplatelet-exposed and unexposed cohorts before propensity score matching. An alpha level of 0.05 was selected for statistical significance

SD: standard deviation

After propensity score-matching, 25,788 patients were identified for both lumbar puncture patient cohorts with and without anticoagulant exposure. Age at index were 47.9 ± 22.0 years and 48.2 ± 22.1 years, respectively. 47.3% and 47.4% were males in the anticoagulant-use and non-anticoagulant-use cohorts, respectively. Post-propensity score-matching baseline characteristics and demographics are shown in Table 3 for the anticoagulant versus non-anticoagulant comparative study. We identified 13,310 lumbar puncture patients for both antiplatelet-exposed and non-antiplatelet-exposed cohorts after propensity score-matching, and their age at index were 58.0 ± 17.8 years and 59.0 ± 18.0 years, respectively. 49.0% of the antiplatelet-exposed cohort and 48.5% of the non-antiplatelet-exposed cohort were males. Post-propensity score-matching baseline characteristics and demographics are shown in Table 4 for the antiplatelet versus non-antiplatelet comparative study.

**Table 3:** Post-propensity score-matching baseline characteristics and demographics of lumbar puncture patients with and without anticoagulant use history

Baseline characteristic	Anticoagulant-exposed	% of cohort	Non-anticoagulant-exposed	% of cohort	*p*-value	Standard mean difference
Patients	25,788	100%	25,788	100%		
Age at Index	47.9 (+/- 22.0 SD)	100%	48.2 (+/- 22.1 SD)	100%		
Male	12,209	47.3%	12,216	47.4%	0.951	0.001
Female	13,577	52.6%	13,567	52.6%	0.930	0.001
White	16,179	62.7%	16,165	62.7%	0.899	0.001
Black or African American	5,173	20.1%	5,444	21.1%	0.003	0.026
Asian	542	2.1%	545	2.1%	0.927	0.001
Unknown Race	3,691	14.3%	3,441	13.3%	0.001	0.028
Fibrosis and Cirrhosis of Liver	493	1.9%	443	1.7%	0.099	0.015
Hypertensive Diseases	9,897	38.4%	9,945	38.6%	0.664	0.004
Diabetes Mellitus	4,432	17.2%	4,420	17.1%	0.889	0.001
Overweight, Obesity, Other Hyperalimentation	3,562	13.8%	3,473	13.5%	0.254	0.010
Nicotine Dependence	4,007	15.5%	4,096	15.9%	0.282	0.009
Alcohol Abuse	1,105	4.3%	1,094	4.2%	0.811	0.002
Alcohol Dependence	1,046	4.1%	966	3.7%	0.069	0.016
Chronic Lower Respiratory Diseases	3,772	14.6%	3,653	14.2%	0.136	0.013
Atrial Fibrillation and Atrial Flutter	1,364	5.3%	1,196	4.6%	0.001	0.030
Heart Failure	1,630	6.3%	1,442	5.6%	< 0.001	0.031
Ischemic Heart Diseases	2,766	10.7%	2,518	9.8%	< 0.001	0.032
Other Peripheral Vascular Diseases	739	2.9%	632	2.5%	0.003	0.026
Thrombocytopenia, Unspecified	1,650	6.4%	1,625	6.3%	0.652	0.004
Immune Thrombocytopenic Purpura	59	0.2%	51	0.2%	0.445	0.007
Qualitative Platelet Defects	40	0.2%	32	0.1%	0.345	0.008
Von Willebrand’s Disease	19	0.1%	19	0.1%	1.00	< 0.001
Hereditary Factor XI Deficiency	10	0.0%	10	0.0%	‘1.00	< 0.001
Disseminated Intravascular Coagulation	123	0.5%	110	0.4%	0.393	0.008
Hereditary Factor VIII Deficiency	10	0.0%	10	0.0%	‘1.00	< 0.001
Hereditary Factor IX Deficiency	10	0.0%	10	0.0%	‘1.00	< 0.001
Activated Protein C Resistance	89	0.3%	79	0.3%	0.440	0.007
Prothrombin Gene Mutation	74	0.3%	65	0.3%	0.445	0.007
Antiphospholipid Syndrome	90	0.3%	75	0.3%	0.242	0.010
Lupus Anticoagulant Syndrome	81	0.3%	72	0.3%	0.466	0.006

This table displays the baseline characteristics and demographics of the patients in the anticoagulant-exposed and unexposed cohorts after propensity score matching. An alpha level of 0.05 was selected for statistical significance

SD: standard deviation

**Table 4:** Post-propensity score-matching baseline characteristics and demographics of lumbar puncture patients with and without antiplatelet use history

Baseline characteristic	Antiplatelet-exposed	% of cohort	Non-antiplatelet-exposed	% of cohort	*p*-Value	Standard mean difference
Patients	13,310	100%	13,310	100%		
Age at Index	58.0 (+/- 17.8 SD)	100%	59.0 (+/- 18.0 SD)	100%	< 0.001	0.056
Male	6,520	49.0%	6,450	48.5%	0.391	0.011
Female	6,789	51.0%	6,855	51.5%	0.418	0.01
White	8,721	65.5%	8,631	64.8%	0.247	0.014
Black or African American	2,577	19.4%	2,689	20.2%	0.085	0.021
Asian	226	1.7%	268	2.0%	0.056	0.023
Unknown Race	1,679	12.6%	1,624	12.2%	0.307	0.013
Fibrosis and Cirrhosis of Liver	320	2.4%	305	2.3%	0.544	0.007
Hypertensive Diseases	7,088	53.3%	7,137	53.6%	0.547	0.007
Diabetes Mellitus	3,449	25.9%	3,396	25.5%	0.457	0.009
Overweight, Obesity, Other Hyperalimentation	2,225	16.7%	2,045	15.4%	0.003	0.037
Nicotine Dependence	2,100	15.8%	2,004	15.1%	0.103	0.020
Alcohol Abuse	614	4.6%	583	4.4%	0.359	0.011
Alcohol Dependence	543	4.1%	543	4.1%	1.00	< 0.001
Chronic Lower Respiratory Diseases	2,385	17.9%	2,249	16.9%	0.028	0.027
Atrial Fibrillation and Atrial Flutter	1,256	9.4%	1,065	8.0%	< 0.001	0.051
Heart Failure	1,510	11.3%	1,315	9.9%	< 0.001	0.048
Ischemic Heart Diseases	2,685	20.2%	2,333	17.5%	< 0.001	0.068
Other Peripheral Vascular Diseases	652	4.9%	561	4.2%	0.007	0.033
Thrombocytopenia, Unspecified	1,072	8.1%	988	7.4%	0.054	0.024
Immune Thrombocytopenic Purpura	39	0.3%	38	0.3%	0.909	0.001
Qualitative Platelet Defects	33	0.2%	25	0.2%	0.293	0.013
Von Willebrand’s Disease	10	0.1%	10	0.1%	1.00	< 0.001
Hereditary Factor XI Deficiency	10	0.1%	0	0%	0.002	0.039
Disseminated Intravascular Coagulation	81	0.6%	68	0.5%	0.286	0.013
Hereditary Factor VIII Deficiency	10	0.1%	10	0.1%	1.00	< 0.001
Hereditary Factor IX Deficiency	10	0.1%	10	0.1%	1.00	< 0.001
Activated Protein C Resistance	70	0.5%	58	0.4%	0.288	0.013
Prothrombin Gene Mutation	60	0.5%	49	0.4%	0.291	0.013
Antiphospholipid Syndrome	73	0.5%	59	0.4%	0.222	0.015
Lupus Anticoagulant Syndrome	66	0.5%	53	0.4%	0.232	0.015

This table displays the baseline characteristics and demographics of the patients in the antiplatelet-exposed and unexposed cohorts after propensity score matching. An alpha level of 0.05 was selected for statistical significance

SD: standard deviation

Table 5 describes the outcomes of subdural and epidural hematomas that occurred within 30 days after undergoing lumbar puncture by comparing the cohorts with and without a history of anticoagulant use. When comparing the occurrence of adverse outcomes for the anticoagulant-exposed cohort to the non-anticoagulant-exposed cohort, epidural hematoma (OR [95%CI], 1.000 [0.561–1.784]) showed no difference in odds ratio between two cohorts with no statistical significance. However, subdural hematoma (OR [95%CI], 0.780 [0.640–0.950]) showed a statistically significantly lower odds ratio for the anticoagulant use group. Table 6 shows the outcomes of subdural and epidural hematomas that occurred within 30 days after undergoing lumbar puncture by comparing the cohorts with and without a history of antiplatelet use. Both subdural hematoma and epidural hematoma outcomes were not statistically significantly increased in one cohort versus another.

**Table 5:** Risk differences, risk ratios, and odds ratios of developing subdural hematoma and epidural hematoma within 30 days after lumbar puncture for patients with and without anticoagulant use history

Outcome	Risk difference	95% CI	*p*-Value	Risk ratio	95% CI	Odds ratio	95% CI
Subdural Hematoma	-0.002	(-0.004, -0.000)	0.013	0.782	(0.643, 0.951)	**0.78**	**(0.640**,** 0.950)**
Epidural Hematoma	0.000	(-0.001, 0.001)	0.999	1.000	(0.561, 1.783)	**1.000**	**(0.561**,** 1.784)**

This table displays the risk differences, risk ratios, and odds ratios comparing various outcomes experienced within 30 days of lumbar puncture by the anticoagulant exposed and unexposed cohorts. An alpha level of 0.05 was selected for statistical significance

CI: confidence interval

**Table 6:** Risk differences, risk ratios, and odds ratios of developing subdural hematoma and epidural hematoma within 30 days after lumbar puncture for patients with and without antiplatelet use history

Outcome	Risk difference	95% CI	*p*-Value	Risk Ratio	95% CI	Odds ratio	95% CI
Subdural Hematoma	0.000	(-0.002, 0.002)	0.952	1.008	(0.785, 1.293)	**1.008**	**(0.784**,** 1.296)**
Epidural Hematoma	0.001	(-0.000, 0.001	0.094	1.902	(0.885, 4.089)	**1.904**	**(0.885**,** 4.095)**

This table displays the risk differences, risk ratios, and odds ratios comparing various outcomes experienced within 30 days of lumbar puncture by the antiplatelet exposed and unexposed cohorts. An alpha level of 0.05 was selected for statistical significance

CI: confidence interval

## Discussion

The present study evaluated the risk of developing subdural hematoma and epidural hematoma after a lumbar puncture in patients with a history of antiplatelet and anticoagulation therapy. Many case reports have shown examples of complications, such as hematomas and bleeding following lumbar punctures, with patients on antiplatelet and anticoagulation therapy. In contrast to these studies, this study offers a multi-institutional view of the risk associated with lumbar puncture while on antiplatelet or anticoagulation therapy. Utilizing a large cohort population, a clearer association can be established between anticoagulation and antiplatelet medication use and the risk of complications associated with lumbar puncture.

### Subdural hematoma and antiplatelets

Based on our results, there is no increased risk of subdural hematoma following lumbar puncture in patients with antiplatelet therapy (OR [95%CI], 1.008 [0.784–1.296]). Similar findings were shown in a prospective cohort study conducted by Horlocker et al., where 924 patients underwent spinal anesthesia on antiplatelet therapy. The study found that minor bleeding complications were increased on antiplatelet therapy, but none of the patients developed spinal hematomas [18]. Additionally, a literature review spanning from 1906 to 1994 conducted by Vandermeulen et al. evaluated bleeding risk following lumbar punctures and found that only 3 out of 61 cases of bleeding occurred in patients with antiplatelet use [19]. While its antiplatelet therapy, such as aspirin, may not increase the risk of hematomas, combination with anticoagulation as well as dual antiplatelet therapy has shown an increased risk [20–22]. This may be of interest for future investigation involving large multi-institutional representative population-level data. Overall, most literature is concurrent with the current study finding that antiplatelet therapy does not significantly increase the risk of subdural hematoma following lumbar puncture.

### Subdural hematoma and anticoagulants

However, in contrast to the existing literature, our study showed a slightly decreased risk of developing a subdural hematoma for anticoagulation use cohort (OR [95%CI], 0.780 [0.640–0.950]). This finding can be best explained by analyzing the existing evidence regarding subdural hematomas following lumbar punctures. In patients with anticoagulation therapy, an increased risk of hematomas and other major bleeding complications has been shown by various studies such as the one by Ruff et al. [20] However, this study initiated anticoagulation after the lumbar puncture and did not explicitly study the risk of hematomas in patients already on anticoagulation therapy, which was the focus of our study. Many case studies and case series have also shown an increased risk of subdural hematoma in patients with anticoagulation [23–25]. However, these studies, specifically those conducted by Ito et al. and Kosarchuk et al., do not include procedural or iatrogenic causes of subdural hematoma but rather spontaneous cases [23, 25]. As a result, comparing our study’s results directly with the referenced study models and criteria poses challenges, and our study, therefore, adds value and a new perspective to the current existing literature.

### Epidural hematoma and antiplatelets

From our study, we found that there was no statistically significant association between the use of antiplatelet therapy and risk for epidural hematoma following lumbar puncture (OR [95%CI], 1.904 [0.885–4.095]). This finding is consistent with two retrospective studies analyzing epidural hematoma formation following spinal anesthesia, an equivalently invasive procedure to lumbar puncture. A study conducted by Horlocker et al. reported that out of a total of 391 patients on preoperative antiplatelet medication, none developed signs of spinal hematoma [26]. The impact of other antiplatelet medications, including thienopyridines such as clopidogrel, ticlopidine, and prasugrel, is largely unclear as only small-sampled case studies show an increased risk of hematoma formation after lumbar puncture [27, 28]. However, our study at a larger scale includes these antiplatelet medications in its analysis, demonstrating that the overall risk of spinal hematoma from lumbar puncture procedure with a history of thienopyridine use is not statistically significant (OR [95%CI], 1.904 [0.885–4.095]). Furthermore, a recent retrospective study investigating aspirin/clopidogrel use and spinal hematoma formation supports our findings [29]. Overall, this study supports the consensus that no statistically significant risk exists for epidural hematoma following lumbar puncture in patients with a history of antiplatelet use.

### Epidural hematoma and anticoagulants

Multiple case studies have shown the development of epidural hematomas following heparin and other anticoagulation use [30, 31]. However, our study found no significant association between the prior use of anticoagulation therapy and risk for epidural hematoma following lumbar puncture (OR [95%CI], 1.000 [0.561–1.784]). Other studies, such as a 10-year retrospective review of central neuraxial blockade complications conducted by Moen et al. in Sweden, reported that spinal hematoma formation may be associated with anticoagulation. However, their data was inconclusive as only one-third of their patients’ outcomes were on anticoagulation [32]. Thus, no clear consensus is seen between lumbar puncture-induced spinal hematoma and anticoagulation, potentially supporting the results of our study, which showed no significant association between the risk and the outcome.

The consensus throughout current literature denotes the feasibility of performing lumbar punctures even with patients who have a prior history of using antiplatelets or anticoagulants. As discussed, the rates of complications, including but not limited to hematomas, are nominal for them to have major clinical relevance unless patients have severe pre-procedural comorbidities that need to be accounted for.

We acknowledge that there are limitations to this study. The major limitation of our study is that it is a retrospective cohort study. Furthermore, due to the limiting nature of the TriNetX database with de-identified patient information, we were not able to get specific patient-based anticoagulant and antiplatelet drug information. In addition, there is some degree of misidentification and misinformation, given the fact that TriNetX is a multi-institutional database. Nonetheless, the population-level adverse outcomes analysis of this retrospective cohort study sheds light on future research where cohorts can be designed with specific pertinent patient information to validate our current findings and further analyze the adverse outcomes of lumbar puncture procedures.

## Conclusion

In summary, while most of our findings are concurrent with the current literature, no all-encompassing explanation exists that covers the entirety of the subject of risk of developing subdural hematoma and epidural hematoma following lumbar puncture for patients who have used anticoagulants or antiplatelets. Similar validation cohort studies will need to be performed to elucidate any correlations between the risk factors and potential adverse outcomes when performing lumbar punctures. This study set the stage for further investigation of the topic with the goal of bettering patient outcomes.

## Data Availability

The data is available upon request.
